# Inhibitory Effects of iPSC-MSCs and Their Extracellular Vesicles on the Onset of Sialadenitis in a Mouse Model of Sjögren's Syndrome

**DOI:** 10.1155/2018/2092315

**Published:** 2018-03-15

**Authors:** Bo Hai, Taeko Shigemoto-Kuroda, Qingguo Zhao, Ryang Hwa Lee, Fei Liu

**Affiliations:** Institute for Regenerative Medicine, Molecular and Cellular Medicine Department, College of Medicine, Texas A&M University Health Science Center, College Station, TX 77843, USA

## Abstract

No effective treatment for Sjögren's syndrome (SS), a chronic autoimmune disease affecting mainly salivary and lacrimal glands, is available now. Systemic infusion of allogeneic mesenchymal stem cells (MSCs) isolated from tissues such as bone marrow (BM) alleviated SS in mouse models and a small clinical trial, but further research and application of this MSC therapy were hindered by limited expandability, significant donor variations, and safety concerns of tissue-derived MSCs. To circumvent these issues, we derived MSCs from human iPSCs using an optimized protocol that can be easily scaled up to produce a huge amount of standardized MSCs. Our iPSC-MSCs inhibited the onset of lymphocyte infiltration into salivary glands in the NOD mouse model of SS in the same way as BM-MSCs. Extracellular vesicles (EVs) carry bioactive molecules in the same way as their originating cells and are more stable and considered much safer than cells for therapies. We found that EVs derived from BM-MSCs and iPSC-MSCs suppressed activation of immune cells and expression of proinflammation factors essential for SS progression in vitro and that infusion of iPSC-MSC EVs at the predisease stage decreased the lymphocyte infiltration in salivary glands and serum autoantibody levels in the same way as infusion of BM-MSCs and iPSC-MSCs. These data suggested that iPSC-MSC EVs have the potential to prevent the progression of SS before the onset of sialadenitis.

## 1. Introduction

Sjögren's syndrome (SS) is a chronic inflammatory autoimmune disease affecting mainly salivary glands (SGs) and lacrimal glands with an incidence of about 1% in the general population and up to 3% in people above the age of 50, with women accounting for more than 90% of diagnosed cases [1]. No effective treatment of SS is available now [2]. Hypofunction of SGs or xerostomia is a major symptom of SS that exacerbates dental caries and periodontal disease; causes problems of mastication, swallowing, speech, and sleep and loss of taste; and hence severely impairs the quality of life of patients. The intravenous (IV) infusion of allogeneic mesenchymal stem/stromal cells (MSCs) isolated from bone marrow (BM) or umbilical cord alleviated experimental and clinical SS [3]. The underlying mechanisms are related to the increase in regulatory T cells (Tregs) expressing IL10 and TGF*β* and the inhibition on differentiation of T follicular helper (Tfh) and T helper 17 (Th17) cells [3, 4]. However, the application of tissue-derived allogeneic MSCs for SS treatment is hindered by their limited expandability, their considerable variations in biological properties caused by donors and different expansion methods, the lack of standard assays for testing therapeutic efficacy of these cells, and safety concerns such as the protumor potential [5]. Particularly, our previous study revealed that MSCs isolated from different donors exhibited a huge variation in their therapeutic efficacy in suppressing inflammation in vivo, and some tissue-derived MSCs even failed to show any therapeutic effects on animal models of sterile inflammation-mediated diseases [6]. To overcome limitations of tissue-derived MSCs, we have derived MSCs efficiently from transgene-free human induced pluripotent stem cells (iPSCs) with almost unlimited expandability using an optimized protocol that can be easily scaled up to produce a huge amount of standardized MSCs [7]. Our iPSC-derived MSCs showed anti-inflammatory properties comparable to those of BM-MSCs in a murine model of corneal injury [8], and iPSC-derived MSCs generated by other groups also showed potent immunosuppressive properties similar to those of tissue-derived MSCs in vitro [9].

In spite of cell sources, there are many other practical difficulties in MSC therapies such as the risk of pulmonary embolism, the need for cryogenic storage/shipping and for thawing cryopreserved cells and the related high cost, and dynamic changes of living MSCs caused by various factors such as thawing, shear stress during injection, and in vivo microenvironments. In the NOD mouse model of SS, IV infusion of soluble intracellular contents from allogeneic bone marrow-derived cells including MSCs (BM soup) before the onset of SS prevented sialadenitis and xerostomia in the same way as IV infusion of BM-MSCs [10], suggesting that cell-free products of MSCs may prevent SS progression in the same way as live MSCs. Recent studies suggest that extracellular vesicles (EVs) derived from MSCs may resolve current problems with cell-based MSC therapies. EVs are membrane-surrounded nanoparticles released from cells spontaneously, carry bioactive molecules in the same way as their originating cells, and mediate many functions of originating cells through enhanced delivery of these molecules into target cells [11]. EVs remain stable and functional for weeks at 4°C and months at −80°C [12] and are generally considered much safer as cell-free products for therapies compared to their originating cells. EVs can be classified as exosomes, microvesicles, and apoptotic bodies based on their biogenesis. However, the dimensions and density of exosomes and microvesicles can overlap, making it technically challenging to analyze their individual properties separately [13], so the general term EV is preferred in more recent literatures and is used here.

MSC EVs show immunomodulatory effects similar to MSCs. In vitro studies indicated that the uptake of MSC-derived EVs occurs in both resting and, mostly, activated B, NK, and T cells and results in the inhibition of proliferation of these immune cells [14]. MSC EVs can also inhibit B cell differentiation and antibody production [15]. Our recent study indicated that IV-injected human BM-MSC EVs effectively prevent the onset of type 1 diabetes (T1D) and experimental autoimmune uveoretinitis (EAU) in mouse models likely by inhibiting the activation of antigen-presenting cells (APCs) and development of T helper 1 (Th1) and Th17 cells [16].

We reported here for the first time that iPSC-MSCs prevented SS progression before the onset of sialadenitis in the same way as BM-MSCs. iPSC-MSC EVs suppressed the activation of immune cells and expression of proinflammation factors essential for SS progression in vitro in the same way as BM-MSC EVs, and infusion of iPSC-MSC EVs at the predisease stage decreased the lymphocyte infiltration in SGs and serum levels of autoantibodies in the same way as infusion of MSCs. These findings suggested that iPSC-MSCs and EVs derived from them are promising to circumvent limitations of tissue-derived MSCs for preventing SS onset.

## 2. Materials and Methods

### 2.1. Culture Conditions of MSCs

The iPSC-MSCs differentiated in our laboratory [7] were plated at a density of 500 cells per cm^2^ of the growth area in *α*MEM medium containing 17% (*v*/*v*) heat-inactivated fetal bovine serum (FBS, Atlanta Biologicals) at 37°C and 5% CO_2_ and split at 70–80% confluence as previously optimized for BM-MSCs [6, 17–19]. The BM-MSCs were acquired from our NIH-funded MSC distribution center (https://medicine.tamhsc.edu/irm/msc-distribution.html) and cultured under the same conditions as iPSC-MSCs. The iPSC-MSCs and BM-MSCs at passage 5 (P5) with 70–80% confluence were harvested for all experiments.

### 2.2. Animal Studies

Female NOD mice (NOD/ShiLtJ) and BALB/c mice were purchased from the Jackson Laboratory and housed in our pathogen-free animal facility. All animal works were approved by the Texas A&M University Institutional Animal Care and Use Committee (IACUC). At 8 and 9 weeks of age, mice were injected via tail vein twice with the vehicle control PBS, 1 × 10^6^ iPSC-MSCs or BM-MSCs, or 30 *μ*g iPSC-MSC EVs. At 12 weeks of age, mice were euthanized to collect SMG and serum samples. One of each pair of SMGs was sectioned for histology evaluation, and the other was used for extraction of total RNAs for qRT-PCR analysis.

### 2.3. Preparation and Characterization of EVs

For EV preparation, BM-MSCs and iPSC-MSCs at 70–80% confluence in 17% FBS *α*MEM medium were transferred to the chemically defined and protein-free medium based on CD-CHO medium (Invitrogen, cat. number 10743-002) as reported [20]. After 6 hr, the medium was discarded and replaced by fresh medium and recovered at 48 hr to isolate EVs. Briefly, the conditioned medium was centrifuged at 2565*g* for 15 min to remove cells and debris, and then EVs were isolated from the supernatant by ultracentrifugation at 100,000*g* for 90 minutes at 4°C using Sorvall WX Floor Ultra Centrifuge with AH-629 36 ml swinging Bucket Rotor (Thermo). EV pellets were dissolved in cold TM buffer (pH 8.6) overnight at 4°C and frozen at −80°C in TM buffer (pH 8.6) containing 1% sucrose and 1% glycerol. The size and concentration of EVs were analyzed using the NanoSight LM 10 Nanoparticle Tracking Analysis System (Malvern). The NanoSight instrument was calibrated with polystyrene latex 100 nm and 200 nm microbeads (NTA4088 and NTA4089). Samples were measured under a range of a particle count from 2 × 10^8^ to 1 × 10^9^ particles per milliliter.

### 2.4. LPS-Stimulated Mouse Lymphocyte Reaction

Mouse splenocytes were isolated from 12-week-old female BALB/c mice (Jackson Laboratory). The splenocytes (3 × 10^5^ cells/well) were incubated with RPMI-1640 medium (ATCC) containing 5% heat-inactivated FBS plus 100 units/ml penicillin and 100 mg/ml streptomycin (pen/strep; both from Life Technologies) in 96-well plates. LPS (Sigma) was added into the culture with a final concentration of 50 ng/ml with or without EVs from BM-MSCs or iPSC-MSCs. One day later, 0.15 ml cell-free supernatant was harvested from each well by centrifugation as mentioned above to measure cytokine expression by ELISA in triplicate as recommended by the manufacturer's protocol, and 3 wells of splenocyte culture were measured for each treatment.

### 2.5. Coculture of Human Lymphocytes with Salivary Gland Epithelial Cells

Human peripheral blood mononuclear cells (PBMCs) from three different donors were purchased from Lonza. Healthy human salivary gland epithelial cells (SGECs) were isolated and cultured as we reported previously [21]. The SGEC-PBMC coculture experiment was based on a published protocol [22]. Briefly, SGECs were seeded at 1 × 10^5^ cells per well into 12-well plates and cultured in Keratinocyte serum-free medium (SFM, Life technology) with poly I:C (5 *μ*g/ml, InvivoGen, tlrl-picw) for 12 hours to allow attachment, stimulation of autoantigen synthesis [23], and IL7 expression essential for SS progression [24]. After removing the SFM and washing with PBS, 2 × 10^4^ PBMCs per well were added in LGM-3 lymphocyte growth medium (Lonza) containing 10% FBS and phytohemagglutinin-P (PHA-P, 5 *μ*g/ml, Sigma-Aldrich, L8754) to activate T cells. After 4 days of coculture, SGECs and PBMCs were harvested separately and analyzed for gene expression by qRT-PCR.

### 2.6. ELISA

The serum levels of autoantibodies anti-La and anti-Ro52 were examined with ELISA kits (Signosis, EA-5203, EA-5204) according to the manufacturer's instructions, and we diluted serum samples at 1 : 5 in the provided diluent buffer for ELISA. Mouse IL12 and IL6 in the culture supernatants of splenocytes were measured by commercial ELISA kits (R&D Systems, Minneapolis, MN) according to the manufacturer's protocol.

### 2.7. Reverse Transcription and Quantitative PCR

Total RNAs were extracted with the RNeasy Plus Mini Kit with on-column DNase I digestion (Qiagen, 74136). Reverse transcription (RT) was carried out with the high-capacity cDNA reverse transcription kit (Thermo Fisher, 4374966). Quantitative PCR (qPCR) was performed with SYBR Green master mix on a 7900HT Fast Real-Time PCR System (Applied Biosystems). The primers were synthesized by Thermo Fisher with sequences retrieved from PrimerBank (https://pga.mgh.harvard.edu/primerbank). PCR assays were run in triplicate with 3 independent samples of each group and analyzed with qBasePlus software (Biogazelle, Belgium) using GAPDH as the reference RNA.

### 2.8. Statistics

All quantified data were analyzed using one-way ANOVA followed by Tukey multiple-comparison test. Statistical analysis and graphical generation of data were done with GraphPad Prism software (San Diego, CA).

## 3. Results

### 3.1. iPSC-MSCs Prevented the Onset of Sialadenitis in the Same Way as BM-MSCs

**Figure 1:**
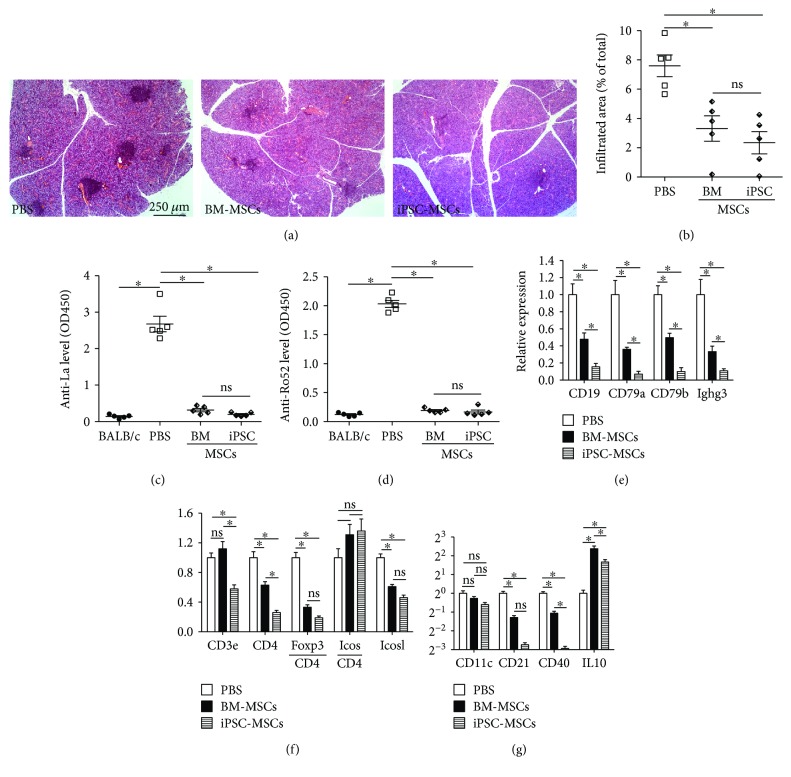
Effects of iPSC-MSC on preventing the onset of sialadenitis in NOD mice. (a) H&E staining of SMG sections. (b) Relative area of lymphocytic infiltrates in SMGs. (c, d) Serum level of autoantibodies determined by ELISA. Expression of genes related to B cells (e), T cells (f), and APCs (g) in SMGs determined by qRT-PCR. *n* = 5; ^∗^*P* < 0.05; ns: not significant.

In the NOD mouse model of SS, lymphocyte infiltration in female SMGs (sialadenitis) begins at 8 weeks of age and is clearly present by 12 weeks of age, whereas the IV infusion of allogeneic mouse BM-MSCs before or at the very beginning of sialadenitis (6 or 8 weeks of age) significantly reduced lymphocytic infiltrates in SMGs for up to 14 weeks after infusion [3, 25]. To determine whether our iPSC-MSCs can similarly prevent the SS progression before the onset of sialadenitis, we injected female NOD mice IV twice at 8 and 9 weeks of age with the vehicle control PBS or 1 × 10^6^ iPSC-MSCs or BM-MSCs and collected SMG and serum samples at 12 weeks of age. H&E staining of SMG sections indicated that in all 5 PBS-treated NOD mice, multiple large lymphocyte infiltrates with >50 cells (focus) [26] were observed (Figures 1(a) and 1(b)), whereas no obvious focus was observed in SMG sections of age- and sex-matched BALB/c mice (data not shown). The size of lymphocyte infiltrates in SMGs in both MSC-treated NOD mice significantly decreased compared to that in the PBS control group (Figures 1(a) and 1(b), *P* < 0.01, *n* = 5), and there was no significant difference between BM-MSC and iPSC-MSC groups (*P* = 0.42). ELISA of serum samples indicated that serum levels of anti-Lo and anti-Ro52 autoantibodies in PBS-treated mice were significantly higher than those in age- and sex-matched BALB/c mice, whereas both types of MSCs significantly decreased serum levels of anti-Lo and anti-Ro52 autoantibodies (Figures 1(c) and 1(d), *P* < 0.005, *n* = 5). To compare the effects of these two types of MSCs on the composition and activation of lymphocytes infiltrated into SMGs, we examined the expression of various lymphocyte markers including B cell marker CD19; B/plasma cell markers CD79a, CD79b, and Ighg3; pan-T cell marker CD3e; helper T cell marker CD4; Treg marker Foxp3; Tfh marker inducible T cell costimulator (Icos) and its ligand Icosl; Th17 marker IL17; pan-APC marker CD11c; follicular dendritic cell (FDC) marker CD21; and activated APC marker CD40, as well as those of immune inhibitory factors including TGF*β*1-3 and IL10 by qRT-PCR. Both MSC treatments significantly decreased the expression of all B/plasma cell markers detected in SMGs with respect to the PBS group, whereas the inhibition effects of iPSC-MSCs were significantly stronger than those of BM-MSCs (Figure 1(e), *P* < 0.05). Both MSC treatments also significantly decreased the expression of CD4, CD4-normalized Foxp3, and Icosl in SMGs, but only iPSC-MSCs significantly decreased CD3e expression, whereas the CD4-normalized Icos level was not significantly affected by either type of MSCs (Figure 1(f)), and IL17 expression was undetectable in all samples (data not shown). The expression of pan-APC marker CD11c was not significantly affected by either type of MSCs, whereas the level of FDC marker CD21 and activated APC marker CD40 was significantly decreased by both MSCs (Figure 1(g)). Both MSC treatments significantly increased IL10 expression (Figure 1(g)) but did not significantly affect the expression of TGF*β*1–3 (data not shown). These data indicated that our iPSC-MSCs prevented SS progression before the onset of sialadenitis with an efficacy comparable to that of BM-MSCs in most indexes and even better in some indexes, which appears mediated by similar inhibitions on the recruitment of B cells and T cell and on the activation of Tfh and APCs, but not mediated by local Tregs in SMGs.

### 3.2. The Physical and Immunomodulatory Properties of iPSC-MSC EVs Are Comparable to Those of BM-MSC EVs

**Figure 2:**
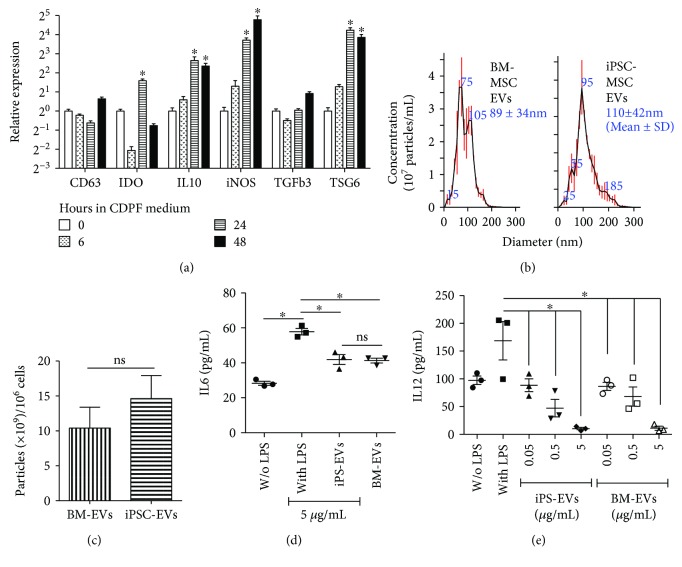
Optimization of iPSC-MSC culture conditions for EV preparation and characterization of EVs. (a) Effects of incubation in CDPF medium on the expression of biomarkers for anti-inflammatory and immunosuppressive properties of MSCs in iPSC-MSCs as determined by qRT-PCR. (b, c) Size distribution and yields of EVs derived from BM-MSCs and iPSC-MSCs measured by NanoSight nanoparticle tracking analysis. (d, e) Effects of two types of MSC EVs on cytokine expression by LPS-stimulated mouse splenocytes as determined by ELISA. *n* = 3; ^∗^*P* < 0.05; ns: not significant.

BM-MSC EVs showed immunosuppressive properties similar to BM-MSCs in vitro [14, 15], and we found recently that human BM-MSC EVs effectively prevented the onset of two other autoimmune diseases in mouse models [16]. Whether EVs derived from iPSC-MSCs possess similar immunomodulatory effects has never been reported. The autoimmune suppressive BM-MSC EVs were prepared from the BM-MSCs cultured under optimized conditions for the production of anti-inflammatory EVs [20], that is, 6–48 hours in chemically defined and protein-free (CDPF) medium. To determine the effects of these culture conditions on our iPSC-MSCs, we examined the mRNA expressions of biomarkers used for the optimization including CD63, TSG6, IL1*β*, and STC1, as well as those of factors related to immunosuppressive effects of MSCs or MSC EVs including IDO [27], iNOS [28], IL10, and TGF*β* [29] by qRT-PCR in iPSC-MSCs cultured for 0, 6, 24, or 48 hours in the CDPF medium. Similar to that reported for BM-MSCs [20], incubating the iPSC-MSCs in CDPF medium for 48 hours significantly upregulated *TSG6* expression and slightly increased *CD63* expression with respect to either the 0- or the 6-hour group (Figure 2(a)), whereas the expressions of *IL1β* and *STC1* were significantly downregulated at all 3 time points after culture in the CDPF medium in iPSC-MSCs in contrast to the reported upregulation in BM-MSCs (data not shown). In addition, 48-hour incubation in the CDPF medium significantly upregulated mRNA expression of IL10 and iNOS and slightly increased *TGFβ3* expression, whereas *IDO* expression was upregulated by 24 but not 48 hours (Figure 2(a)). At all 3 time points after culture in the CDPF medium, *TGFβ1* expression was not significantly affected, whereas *TGFβ2* expression was significantly downregulated (data not shown). These data suggested that culture conditions optimized for the production of anti-inflammatory and immunosuppressive EVs from BM-MSCs are good for preparing similar EVs from iPSC-MSCs. Using the same optimized conditions, we prepared EVs from supernatants of BM-MSCs and our iPSC-MSCs by ultracentrifugation as reported [20]. Both the sizes and yields of EVs from these two types of MSCs were comparable with no significant differences (Figures 2(b) and 2(c), *P* = 0.54 for sizes and *P* = 0.21 for yields, *n* = 3). LPS can activate multiple types of immune cells among mouse splenocytes to secret proinflammatory factors including IL6 and IL12, two proinflammation cytokines essential for SS progression and highly produced by activated helper T cells and APCs, respectively [30, 31], as indicated by ELISA (Figures 2(d) and 2(e), *P* < 0.05, *n* = 3). EVs produced by both BM-MSCs (BM-EVs) and iPSC-MSCs (iPS-EVs) significantly suppressed the induction of IL12 at concentrations from 0.05 to 5 *μ*g/ml in a dose-dependent manner and of IL6 only at the highest concentration of 5 *μ*g/ml in LPS-stimulated mouse splenocytes (Figures 2(d) and 2(e), *P* < 0.05, *n* = 3), and the inhibitory efficacies of these two types of EVs on both cytokines were comparable. These data indicated that physical and immunomodulatory properties are comparable between BM-MSC EVs and iPSC-MSC EVs prepared under optimized conditions mentioned above.

### 3.3. iPSC-MSC EVs Inhibited Interactions between Immune Cells and SGECs in the Same Way as BM-MSC EVs

**Figure 3:**
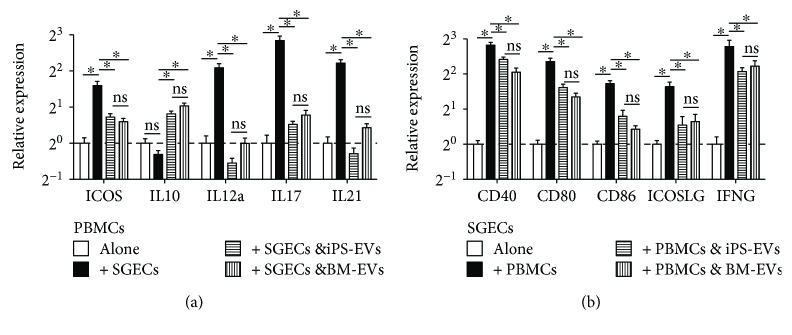
Effects of MSC EVs on gene expression of immune cells and SGECs after coculture. Human PBMCs and SGECs were cocultured at the ratio of 1 : 1 for 3 days in the presence of 2.5 *μ*g/ml phytohemagglutinin with or without EVs derived from BM-MSCs or iPSC-MSCs (5 *μ*g/ml) and then examined for gene expressions in isolated PBMCs (a) and SGECs (b) by qRT-PCR. *n* = 3; ^∗^*P* < 0.05.

Interactions between SG epithelial cells (SGECs) and immune cells are essential for SS progression through multiple mechanisms. SGECs of SS patients can present autoantigens and provide costimulatory signals such as CD40, CD80, and CD86 to activate T cells [32] and induce differentiation of naïve CD4^+^ T cells into Tfh expressing ICOS and IL21 through ICOSLG and IL6 [22]. On the other hand, infiltrated lymphocytes can stimulate SGECs to produce many proinflammation cytokines such as IFN*γ*, IL12, and IL18 [33, 34]. Using a coculture system of human SGECs and PBMCs as reported [22], we examined markers of Tfh, Treg, Th17, and activated APCs in PBMCs and costimulatory molecules and proinflammation cytokines mentioned above in SGECs with or without coculture by qRT-PCR. We confirmed that the coculture induced upregulation of Tfh markers ICOS and IL21 in PBMCs compared to PBMCs cultured alone with the same conditions as reported [22] (Figure 3(a)). Meanwhile, we found that coculture upregulated the mRNA levels of IL12A and IL17A, markers for activated APCs and Th17, respectively, in PBMCs (Figure 3(a)) and mRNA levels of CD40, CD80, CD86, ICOSLG, and IFN*γ* in SGECs (Figure 3(b)). EVs derived from both BM-MSCs and iPSC-MSCs significantly inhibited the upregulation of these genes in PBMCs and SGECs, respectively, and increased the expression of IL10 mRNA in PBMCs (Figure 3(a)). We did not find a significant difference between the effects of BM-MSC EVs and iPSC-MSC EVs on the expression of these genes. The mRNA levels of Treg marker Foxp3 in PBMCs and those of IL6, IL12A, and IL18 in SGECs were not significantly affected by either EV (data not shown), and ICOSLG mRNA expression was undetectable in PBMCs with or without coculture with SGECs, suggesting that the upregulation of IL10 expression by EVs might not be due to the induction of Treg and that the inhibition of Tfh differentiation by MSC EVs is mainly mediated by the repression of ICOSLG expression in SGECs. These data indicated that EVs from both BM-MSCs and iPSC-MSCs can inhibit differentiation of Tfh and Th17 cells, activation of APCs, and expression of costimulatory proteins and proinflammation factors by SGECs induced by interactions between SGECs and immune cells with comparable efficacies.

### 3.4. iPSC-MSC EVs Prevented the Onset of Sialadenitis in the Same Way as MSCs

**Figure 4:**
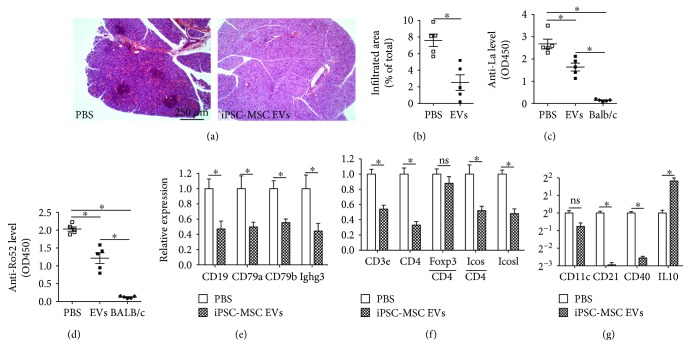
Effects of iPSC-MSC EVs on preventing the onset of sialadenitis in NOD mice. (a) H&E staining of SMG sections. (b) Relative area of lymphocytic infiltrates in SMGs. (c, d) Serum level of autoantibodies determined by ELISA. Expression of genes related to B cells (e), T cells (f), and APCs (g) in SMGs determined by qRT-PCR. *n* = 5; ^∗^*P* < 0.05; ns: not significant.

To evaluate the in vivo effects of iPSC-MSC EVs on SS progression, we injected PBS or iPSC-MSC EVs (30 *μ*g/mouse) into female NOD mice IV twice at 8 and 9 weeks of age based on our recent study of BM-MSC EVs in mouse models of T1D and EAU [16]. At 12 weeks of age, the size of lymphocyte infiltrates in SMGs, serum levels of anti-La/Ro autoantibodies, and the expression of B cell-related genes in SMGs were all significantly decreased in the EV group compared to the PBS control group (Figures 4(a)–4(e), *P* < 0.05, *n* = 5). Compared to iPSC-MSC treatment, iPSC-MSC EV treatment resulted in a comparable inhibition of lymphocyte infiltration in SMGs but significantly less decreases in the expression of B cell-related genes in SMGs and serum autoantibodies (*P* < 0.05). In addition, iPSC-MSC EVs significantly decreased the expression of CD3e, CD4, CD4-normalized Icos, and Icosl in SMGs, but not that of CD4-normalized Foxp3 level (Figure 4(f)). Similar to MSCs, iPSC-MSC EVs did not significantly affect CD11c expression in SMGs but significantly decreased the expression of CD21 and CD40 and increased the IL10 expression (Figure 4(g)). These data indicated that MSC EVs prevent the onset of sialadenitis in the same way as MSCs, whereas the dosage of iPSC-MSC EVs needs to be further optimized to achieve efficacies comparable to those of MSCs for all indexes.

## 4. Discussion

Recent advances in identifying pre-SS patients have opened up the opportunity to prevent SS progression. Many nonsicca (nondryness) features might appear up to 20 years before the development of sicca symptoms in SS patients, including annular erythema, autoimmune cytopenias, or congenital heart block in babies carried by mothers with anti-Ro/SSA antibodies [35]. In patients with primary SS who were autoantibody-positive after diagnosis, autoantibodies such as anti-Ro/La were detected in 66%–81% of patients 4~5 years in median before diagnosis [36, 37], suggesting that the presence of autoantibodies can be used for the early diagnosis of SS to prevent the main sicca symptoms.

Tissue-derived allogeneic MSCs, including those from BM and umbilical cord, alleviated experimental and clinical SS [3], but the donor variation and limited expandability of these tissue-derived MSCs hindered their application. Here, we firstly confirmed that our highly standardized iPSC-MSCs with almost unlimited supply prevented the SS progression in the NOD mouse model with efficacies comparable to those of BM-MSCs. SS pathogenesis relies on interactions between SGECs, APCs, and T cells. SGECs of SS patients serve as atypical APCs to present autoantigens and provide costimulatory signals such as CD40, CD80, and CD86 [32] and induce differentiation of naïve CD4^+^ T cells into Tfh to enhance B cell survival [22]. Professional APCs such as dendritic cells and macrophages are also recruited into SGs of SS patients and contribute to SS progression [38, 39]. The interaction between CD40 on APCs and CD40 ligand (CD40L) on activated T cells is required for the SS onset in the NOD.H-2h4 mouse model [40, 41]. Analyses of SMG tissues suggested that both BM-MSCs and iPSC-MSCs inhibited activations of APCs and Tfh cells likely by downregulating local mRNA expression of ICOSL and CD40. We also found that both MSCs increased mRNA expression of IL10 in SMGs. Although IL10 is considered proinflammatory in SS [42, 43] likely by promoting survival, proliferation, and antibody production of B cells [44], it generally inhibits the maturation and function of APCs and also directly suppresses the differentiation of proinflammatory Th1 and Th17 subsets [45]; hence, its pathogenic effects on SS likely depend on the stage of disease and cell types producing IL10 as indicated by researches in other autoimmune diseases [46, 47]. In our SMG samples, both MSCs significantly decreased lymphocyte infiltration and the expression of marker genes for B/plasma cells, Th cells, and activated APCs but did not significantly affect the expression of pan-APC marker CD11c, suggesting that IL10 at MSC-treated SMGs might mainly inhibit SS progression by repressing the activation of APCs.

Compared to allogeneic MSCs, EVs derived from them appear much safer and more feasible for clinical application due to their superior stability and related low cost of storage, transport, and recovery. BM-MSC EVs have shown immunomodulatory effects similar to BM-MSCs [48, 49] and are promising to inhibit progression of autoimmune responses [16], but no research has been reported on immunomodulatory effects of iPSC-MSC EVs. So we first determined the culture conditions for the production of immunosuppressive EVs from iPSC-MSCs based on previous studies on BM-MSCs and then confirmed that our iPSC-MSC EVs can inhibit expression of proinflammation cytokines essential for SS progression in LPS-stimulated mouse splenocytes with efficacies comparable to those of BM-MSC EVs. Using a coculture system of human SGECs and PBMCs, we found that EVs from both BM-MSCs and iPSC-MSCs inhibited interactions between SGECs and immune cells essential for SS progression, including differentiation of Tfh and Th17 cells and activation of APCs including SGECs. Using the same NOD mouse model, we confirmed that iPSC-MSC EVs significantly inhibited lymphocyte infiltration in SMGs and production of autoantibodies, whereas the efficacies were relatively weaker compared to those of either BM-MSCs or iPSC-MSCs, suggesting the need for further optimization of EV dosage. Similar to both MSCs, iPSC-MSC EVs inhibited activations of APCs and Tfh cells in SMGs likely by downregulating ICOSL and CD40 and also upregulated IL10 expression in SMGs. Human BM-MSCs can inhibit T cell activation by inducing IL10-expressing tolerogenic APCs independently of Tregs [50], and we reported recently that human BM-MSC EVs inhibited activation of mouse APCs in the allogeneic mixed lymphocyte reaction assays [16], suggesting that the induction of IL10-expressing tolerogenic APCs might also contribute to the immunosuppressive effects of iPSC-MSC EVs.

## 5. Conclusion

iPSC-MSCs and EVs derived from them are promising to prevent the progression of SS before the onset of sialadenitis with efficacies comparable to those of BM-MSCs, and the underlying mechanisms are related to inhibitions on the differentiation of Tfh and Th17 cells and the activation of APCs.

## References

[1] Saraux A., Pers J.-O., Devauchelle-Pensec V. (2016). Treatment of primary Sjögren syndrome. *Nature Reviews Rheumatology*.

[2] Brito-Zerón P., Baldini C., Bootsma H. (2016). Sjögren syndrome. *Nature Reviews Disease Primers*.

[3] Xu J., Wang D., Liu D. (2012). Allogeneic mesenchymal stem cell treatment alleviates experimental and clinical Sjögren syndrome. *Blood*.

[4] Liu R., Su D., Zhou M., Feng X., Li X., Sun L. (2015). Umbilical cord mesenchymal stem cells inhibit the differentiation of circulating T follicular helper cells in patients with primary Sjögren’s syndrome through the secretion of indoleamine 2,3-dioxygenase. *Rheumatology*.

[5] Prockop D. J. (2017). The exciting prospects of new therapies with mesenchymal stromal cells. *Cytotherapy*.

[6] Lee R. H., Yu J. M., Foskett A. M. (2014). TSG-6 as a biomarker to predict efficacy of human mesenchymal stem/progenitor cells (hMSCs) in modulating sterile inflammation in vivo. *Proceedings of the National Academy of Sciences of the United States of America*.

[7] Zhao Q., Gregory C. A., Lee R. H. (2015). MSCs derived from iPSCs with a modified protocol are tumor-tropic but have much less potential to promote tumors than bone marrow MSCs. *Proceedings of the National Academy of Sciences of the United States of America*.

[8] Yun Y. I., Park S. Y., Lee H. J. (2017). Comparison of the anti-inflammatory effects of induced pluripotent stem cell–derived and bone marrow–derived mesenchymal stromal cells in a murine model of corneal injury. *Cytotherapy*.

[9] Ng J., Hynes K., White G. (2016). Immunomodulatory properties of induced pluripotent stem cell-derived mesenchymal cells. *Journal of Cellular Biochemistry*.

[10] Misuno K., Tran S. D., Khalili S., Huang J., Liu Y., Hu S. (2014). Quantitative analysis of protein and gene expression in salivary glands of Sjogren's-like disease NOD mice treated by bone marrow soup. *PLoS One*.

[11] Cosenza S., Ruiz M., Maumus M., Jorgensen C., Noël D. (2017). Pathogenic or therapeutic extracellular vesicles in rheumatic diseases: role of mesenchymal stem cell-derived vesicles. *International Journal of Molecular Sciences*.

[12] Yuan Z., Kolluri K. K., Gowers K. H. C., Janes S. M. (2017). TRAIL delivery by MSC-derived extracellular vesicles is an effective anticancer therapy. *Journal of Extracellular Vesicles*.

[13] el Andaloussi S., Mäger I., Breakefield X. O., Wood M. J. A. (2013). Extracellular vesicles: biology and emerging therapeutic opportunities. *Nature Reviews Drug Discovery*.

[14] Di Trapani M., Bassi G., Midolo M. (2016). Differential and transferable modulatory effects of mesenchymal stromal cell-derived extracellular vesicles on T, B and NK cell functions. *Scientific Reports*.

[15] Budoni M., Fierabracci A., Luciano R., Petrini S., di Ciommo V., Muraca M. (2013). The immunosuppressive effect of mesenchymal stromal cells on B lymphocytes is mediated by membrane vesicles. *Cell Transplantation*.

[16] Shigemoto-Kuroda T., Oh J. Y., Kim D. K. (2017). MSC-derived extracellular vesicles attenuate immune responses in two autoimmune murine models: type 1 diabetes and uveoretinitis. *Stem Cell Reports*.

[17] Lee R. H., Pulin A. A., Seo M. J. (2009). Intravenous hMSCs improve myocardial infarction in mice because cells embolized in lung are activated to secrete the anti-inflammatory protein TSG-6. *Cell Stem Cell*.

[18] Bartosh T. J., Ylostalo J. H., Mohammadipoor A. (2010). Aggregation of human mesenchymal stromal cells (MSCs) into 3D spheroids enhances their antiinflammatory properties. *Proceedings of the National Academy of Sciences of the United States of America*.

[19] Lee R. H., Yoon N., Reneau J. C., Prockop D. J. (2012). Preactivation of human MSCs with TNF-*α* enhances tumor-suppressive activity. *Cell Stem Cell*.

[20] Kim D. K., Nishida H., An S. Y., Shetty A. K., Bartosh T. J., Prockop D. J. (2016). Chromatographically isolated CD63^+^CD81^+^ extracellular vesicles from mesenchymal stromal cells rescue cognitive impairments after TBI. *Proceedings of the National Academy of Sciences of the United States of America*.

[21] Hai B., Qin L., Yang Z. (2014). Transient activation of hedgehog pathway rescued irradiation-induced hyposalivation by preserving salivary stem/progenitor cells and parasympathetic innervation. *Clinical Cancer Research*.

[22] Gong Y. Z., Nititham J., Taylor K. (2014). Differentiation of follicular helper T cells by salivary gland epithelial cells in primary Sjögren’s syndrome. *Journal of Autoimmunity*.

[23] Kyriakidis N. C., Kapsogeorgou E. K., Gourzi V. C., Konsta O. D., Baltatzis G. E., Tzioufas A. G. (2014). Toll-like receptor 3 stimulation promotes Ro52/TRIM21 synthesis and nuclear redistribution in salivary gland epithelial cells, partially via type I interferon pathway. *Clinical & Experimental Immunology*.

[24] Jin J. O., Shinohara Y., Yu Q. (2013). Innate immune signaling induces interleukin-7 production from salivary gland cells and accelerates the development of primary Sjögren’s syndrome in a mouse model. *PLoS One*.

[25] Khalili S., Liu Y., Kornete M. (2012). Mesenchymal stromal cells improve salivary function and reduce lymphocytic infiltrates in mice with Sjögren’s-like disease. *PLoS One*.

[26] Szabo K., Papp G., Dezso B., Zeher M. (2014). The histopathology of labial salivary glands in primary Sjögren’s syndrome: focusing on follicular helper T cells in the inflammatory infiltrates. *Mediators of Inflammation*.

[27] Ren G., Su J., Zhang L. (2009). Species variation in the mechanisms of mesenchymal stem cell-mediated immunosuppression. *Stem Cells*.

[28] Sato K., Ozaki K., Oh I. (2007). Nitric oxide plays a critical role in suppression of T-cell proliferation by mesenchymal stem cells. *Blood*.

[29] Bruno S., Deregibus M. C., Camussi G. (2015). The secretome of mesenchymal stromal cells: role of extracellular vesicles in immunomodulation. *Immunology Letters*.

[30] Yoshimoto K., Tanaka M., Kojima M. (2011). Regulatory mechanisms for the production of BAFF and IL-6 are impaired in monocytes of patients of primary Sjögren’s syndrome. *Arthritis Research & Therapy*.

[31] Nocturne G., Mariette X. (2013). Advances in understanding the pathogenesis of primary Sjögren’s syndrome. *Nature Reviews Rheumatology*.

[32] Hughes C. E., Benson R. A., Bedaj M., Maffia P. (2016). Antigen-presenting cells and antigen presentation in tertiary lymphoid organs. *Frontiers in Immunology*.

[33] Sisto M., Lorusso L., Lisi S. (2016). Interleukin-15 as a potential new target in Sjögren’s syndrome-associated inflammation. *Pathology*.

[34] Sakai A., Sugawara Y., Kuroishi T., Sasano T., Sugawara S. (2008). Identification of IL-18 and Th17 cells in salivary glands of patients with Sjögren’s syndrome, and amplification of IL-17-mediated secretion of inflammatory cytokines from salivary gland cells by IL-18. *The Journal of Immunology*.

[35] Brito-Zerón P., Izmirly P. M., Ramos-Casals M., Buyon J. P., Khamashta M. A. (2015). The clinical spectrum of autoimmune congenital heart block. *Nature Reviews Rheumatology*.

[36] Jonsson R., Theander E., Sjöström B., Brokstad K., Henriksson G. (2013). Autoantibodies present before symptom onset in primary Sjögren syndrome. *JAMA*.

[37] Theander E., Jonsson R., Sjöström B., Brokstad K., Olsson P., Henriksson G. (2015). Prediction of Sjögren’s syndrome years before diagnosis and identification of patients with early onset and severe disease course by autoantibody profiling. *Arthritis & Rhematology*.

[38] Gottenberg J. E., Cagnard N., Lucchesi C. (2006). Activation of IFN pathways and plasmacytoid dendritic cell recruitment in target organs of primary Sjögren’s syndrome. *Proceedings of the National Academy of Sciences of the United States of America*.

[39] Christodoulou M. I., Kapsogeorgou E. K., Moutsopoulos H. M. (2010). Characteristics of the minor salivary gland infiltrates in Sjögren’s syndrome. *Journal of Autoimmunity*.

[40] Elgueta R., Benson M. J., de Vries V. C., Wasiuk A., Guo Y., Noelle R. J. (2009). Molecular mechanism and function of CD40/CD40L engagement in the immune system. *Immunological Reviews*.

[41] Mahmoud T. I., Wang J., Karnell J. L. (2016). Autoimmune manifestations in aged mice arise from early-life immune dysregulation. *Science Translational Medicine*.

[42] Perrier S., Serre A. F., Dubost J. J. (2000). Increased serum levels of interleukin 10 in Sjögren’s syndrome; correlation with increased IgG1. *The Journal of Rheumatology*.

[43] Rajagopalan G., Kudva Y. C., Sen M. M. (2006). IL-10-deficiency unmasks unique immune system defects and reveals differential regulation of organ-specific autoimmunity in non-obese diabetic mice. *Cytokine*.

[44] Moore K. W., de Waal Malefyt R., Coffman R. L., O'Garra A. (2001). Interleukin-10 and the interleukin-10 receptor. *Annual Review of Immunology*.

[45] Brooks D. G., Walsh K. B., Elsaesser H., Oldstone M. B. A. (2010). IL-10 directly suppresses CD4 but not CD8 T cell effector and memory responses following acute viral infection. *Proceedings of the National Academy of Sciences of the United States of America*.

[46] Balasa B., la Cava A., van Gunst K. (2000). A mechanism for IL-10-mediated diabetes in the nonobese diabetic (NOD) mouse: ICAM-1 deficiency blocks accelerated diabetes. *The Journal of Immunology*.

[47] Sharif M. N., Tassiulas I., Hu Y., Mecklenbrauker I., Tarakhovsky A., Ivashkiv L. B. (2004). IFN-*α* priming results in a gain of proinflammatory function by IL-10: implications for systemic lupus erythematosus pathogenesis. *The Journal of Immunology*.

[48] Burrello J., Monticone S., Gai C., Gomez Y., Kholia S., Camussi G. (2016). Stem cell-derived extracellular vesicles and immune-modulation. *Frontiers in Cell and Development Biology*.

[49] Favaro E., Carpanetto A., Caorsi C. (2016). Human mesenchymal stem cells and derived extracellular vesicles induce regulatory dendritic cells in type 1 diabetic patients. *Diabetologia*.

[50] Ko J. H., Lee H. J., Jeong H. J. (2016). Mesenchymal stem/stromal cells precondition lung monocytes/macrophages to produce tolerance against allo- and autoimmunity in the eye. *Proceedings of the National Academy of Sciences of the United States of America*.

